# Dementia as a risk factor for 30-day mortality in community-acquired pneumonia in Colombia (CAP): A survival study

**DOI:** 10.1371/journal.pone.0332713

**Published:** 2025-10-03

**Authors:** Juan Sebastian Hernández-Puentes, Alirio Rodrigo Bastidas-Goyes, Eduardo Tuta-Quintero, Diana Díaz-Quijano, Nicole Méndez Peñalosa, Nayah Zuleta Sanchez, María Herran Perez, Juan Rodríguez Sánchez, Valentina Ortiz Marquez, Ana Colmenares Leal, Juanita Zuleta Montañez, Laura Riveros Pedraza, Laura Sarmiento Cardozo, María Piñeros Clavijo, Maria Vasquez Bazurto, Isabella Lenhardt Guaqueta

**Affiliations:** 1 School of Medicine, Universidad de La Sabana, Chía, Colombia; 2 Director of Clinical Medicine Applied Research Group, Chía, Colombia; 3 Department of Epidemiology, Universidad de La Sabana, Chía, Colombia; 4 Department of Internal Medicine, Universidad de La Sabana, Chía, Colombia; University of Bergen: Universitetet i Bergen, NORWAY

## Abstract

**Background:**

Community-acquired pneumonia (CAP) represents a significant clinical challenge, particularly in patients with comorbidities, such as dementia, which increases the risk of mortality and hospitalization. Despite its relevance, dementia has not been considered a CAP severity assessment tool. Furthermore, there is limited information on this topic in Latin America. Therefore, this study aimed to analyze 30-day survival in patients with CAP and dementia in Colombia with the objective of identifying the impact of dementia on clinical outcomes and understanding this phenomenon in the region.

**Methods:**

A multicenter retrospective cohort study with survival analysis was conducted on patients with CAP admitted to two tertiary care institutions in Colombia between January 2010 and December 2020.

**Results:**

The study included 3,374 subjects: 354 (10.5%) with dementia and 3,020 (89.5%) without dementia. The mean age was 65.5 years, higher in the dementia group (82.5 vs. 63.5 years, p < 0.001). Patients with dementia showed a higher prevalence of cyanosis, retraction, altered consciousness, and rales (p < 0.001). They also had higher rates of hypertension, heart failure, cerebrovascular disease, and chronic kidney disease (p < 0.001). Laboratory findings showed lower hemoglobin and sodium levels and higher BUN levels (p < 0.001). The 30-day mortality was 26.3% in the dementia group and 7.1% in the non-dementia group (p < 0.001), with a HR of 2.28 (95% CI: 1.756–2.962). The 30-day survival rate was 73.7% and 92.9%, respectively (p < 0.001).

**Conclusion:**

Patients with dementia who developed pneumonia had a significantly higher mortality rate. They were also characterized by advanced age, multiple comorbidities, and greater disease severity. Our findings emphasize the importance of prioritizing individualized care to improve clinical outcomes and quality of life.

## Introduction

Community-acquired pneumonia (CAP) presents a significant clinical challenge, particularly in patients with comorbidities that substantially influence their evolution and prognosis [1]. Among these, dementia is a crucial factor owing to its high prevalence and increased susceptibility of these patients to respiratory infections [2,3]. CAP is the leading cause of death and hospitalization in individuals with dementia [4]. Moreover, this population group faces up to twice the risk of dying from pneumonia compared to those without this condition (OR = 2.22, 95% CI: 1.44–3.42, p < 0.001) [5].

Graversen et al. [6], in a study based on a cohort of 298,872 patients with CAP, analyzed 30-day post-discharge mortality and readmission rates. The findings revealed that patients with dementia had a 129% higher mortality risk and 7% higher readmission risk than those without dementia. Similarly, another study highlighted that in-hospital mortality was higher in patients aged ≥ 75 years and in those with conditions such as dementia, noting that the prevalence of different pathogens and frailty levels varies by age [7]. These results underscore the importance of considering dementia when assessing the mortality risk and designing personalized treatment strategies.

Despite its clinical relevance, dementia faces significant limitations when considering the tools used to evaluate CAP severity. For instance, the Pneumonia Severity Index (PSI) does not include this condition among its predictors [8]. In addition, information on this topic in the region is scarce. According to a meta-analysis [2], pneumonia-associated mortality in patients with dementia varies considerably depending on the data source and study context, with evidence predominantly from European, American, and Asian populations. This gap limits the applicability of these results to other vulnerable populations, particularly in regions such as Latin America, where research on CAP in patients with dementia remains insufficient [9].

Given the lack of regional data and our aim to characterize this phenomenon in our population, we conducted a 30-day survival analysis in patients with CAP admitted to tertiary care institutions in Colombia. This study aimed to identify the impact of dementia on the clinical course of CAP and to provide relevant data to optimize therapeutic and predictive strategies in our region.

## Methods

A multicenter retrospective cohort study with survival analysis was conducted on patients with CAP admitted to two tertiary care institutions in Colombia between January 2010 and December 2020.

### Eligibility criteria

The study included adults over 18 years of age diagnosed with pneumonia according to the American Thoracic Society/Infectious Diseases Society of America (ATS/IDSA) criteria [10]. Eligible patients presented with acute respiratory symptoms (cough, dyspnea, fever, pleuritic pain, and/or altered mental status) associated with radiological findings compatible with pneumonia (alveolar and/or interstitial opacities, presence of unilateral or bilateral pulmonary consolidations), and requiring antibiotic management. Patients were excluded if they lacked medical records, had incomplete PSI score variables [8], or were diagnosed with nosocomial pneumonia during hospitalization.

### Variables

Data on demographic characteristics, comorbidities, vital signs, physical examination findings, laboratory parameters, arterial blood gases, diagnostic imaging, clinical symptom progression at admission, and treatment were collected. Patients diagnosed with dementia were included with 30-day all-cause mortality as the primary outcome variable.

To minimize transcription bias, data were verified by at least two members of the research team directly from electronic medical records, and data collection personnel received the appropriate training.

### Sample size

The sample size was calculated using the method described by Ahnn and Anderson [11] based on the log-rank test to compare survival curves. Previous data from a study by Zuliani et al. [12] showed hospital mortality rates of 24.3% and 9.7% in patients with and without dementia, respectively. Assuming a 95% confidence level, 90% power, estimated loss of 10%, and a sample-size ratio of 3:1, a minimum sample size of 288 participants was determined.

### Data analysis

Data were extracted directly from electronic medical records, which were reviewed in their entirety and recorded using Research Electronic Data Capture (REDCap) electronic data capture software. Subsequently, the data were accessed for research purposes between 17/10/2024 and 01/12/2024, and then extracted and exported to Excel for final analysis using the Stata 14 software licensed by Universidad de La Sabana. Qualitative variables were summarized as counts and percentages, whereas quantitative variables were expressed as means and standard deviations for normal distributions or medians and interquartile ranges for non-normal distributions. Normality tests were performed using the Kolmogorov-Smirnov test. For independent sample analyses, the two-sample Student’s t-test with Welch correction and Mann-Whitney U test were employed.

Survival analysis was conducted using the Kaplan-Meier method to estimate survival curves for patients with and without dementia who had CAP. Differences between the curves were evaluated using the log-rank test. Multivariate analysis was performed using Cox proportional hazards to identify potential confounders for 30-day mortality in the general pneumonia population, controlling for relevant demographic and clinical variables with their respective hazard ratios (HR) and 95% confidence intervals. Statistical significance was set at p < 0.05.

### Ethical considerations

This study considered minimal risk according to Law 8430 of 1993 [13]. All privacy policies were respected.

## Results

**Fig 1:**
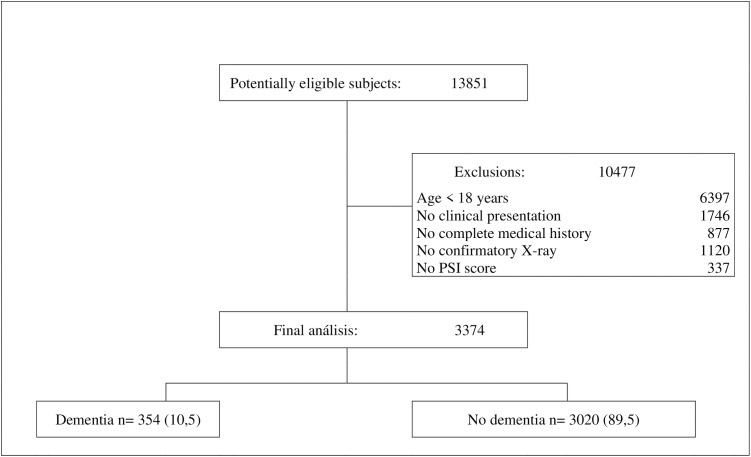
Flowchart of study exclusion and inclusion criteria, Pneumonia Severity Index (PSI).

After applying the inclusion and exclusion criteria from the original database of 13,851 patients, a final sample of 3,374 subjects was obtained, with 354 (10.5%) in the dementia group and 3,020 (89.5%) in the non-dementia group (Fig 1).

### Population characteristics

The mean age of the total study population had a mean age of 65.5 years (SD: 21.44) (Table 1). Patients with dementia were older, with a mean age of 82.5 years (SD: 10.94), than those without dementia at 63.5 years (SD: 21.49; p < 0.001). The proportion of males in the dementia group was 53.7% (p = 0.014). Regarding clinical symptoms, patients with and without dementia showed different prevalence rates of cyanosis (13.6% vs. 7.2%; p < 0.001), retractions (41.2% vs. 23.4%; p < 0.001), altered consciousness (38.1% vs. 10.3%; p < 0.001), and crackles (70.6% vs. 55.2%; p < 0.001).

**Table 1:** General characteristics of the Population.

	Total Population n = 3374	Dementia n = 354	No Dementia n = 3020	p Value
Age years, m (SD)	65,5 (21,44)	82,5 (10,94)	63,5 (21,49)	<0,001
Male sex, n (%)	2016 (59,8)	190 (53,7)	1826 (60,5)	0,014
**Clinical symptoms, n (%)**				
Cyanosis	265 (7,9)	48 (13,6)	217 (7,2)	<0,001
Intercostal retractions	854 (25,3)	146 (41,2)	708 (23,4)	<0,001
Cough	2801 (83)	283 (79,9)	2518 (83,4)	0,104
Altered state of consciousness	447 (13,2)	135 (38,1)	312 (10,3)	<0,001
Crackles	1917 (56,8)	250 (70,6)	1667 (55,2)	<0,001
**Vital signs, m (SD)**				
Heart rate	91,3 (18,68)	88,3 (20,26)	91,7 (18,46)	<0,001
Mean Arterial Pressure, mmHg	88,9 (14,89)	86,4 (16,81)	89,2 (14,62)	<0,001
Body temperature °C	36,8 (0,88)	36,7 (0,77)	36,8 (0,9)	<0,001
Glasgow Coma Scale	14,6 (1,35)	13,9 (1,9)	14,7 (1,21)	<0,001
Oxygen saturation	89 (6,94)	88,3 (6,79)	89,1 (6,95)	<0,001
FiO2	26,1 (10,77)	28,1 (10,96)	25,8 (10,72)	<0,001
**Medical history, n (%)**				
Hypertension	1689 (50,1)	233 (65,8)	1456 (48,2)	<0,001
Smoking	735 (21,8)	108 (30,5)	627 (20,8)	<0,001
Chronic heart failure	517 (15,3)	94 (26,6)	423 (14)	<0,001
Acute myocardial infarction	191 (5,7)	33 (9,3)	158 (5,2)	0,002
Peripheral vascular disease	108 (3,2)	20 (5,6)	88 (2,9)	0,006
Cerebrovascular disease	285 (8,4)	74 (20,9)	211 (7)	<0,001
Ulcer disease	45 (1,3)	10 (2,8)	35 (1,2)	0,010
Liver disease	16 (0,5)	5 (1,4)	11 (0,4)	0,007
Diabetes mellitus	418 (12,4)	57 (16,1)	361 (12)	0,025
Chronic kidney disease	173 (5,1)	42 (11,9)	131 (4,3)	<0,001
Hemiplegia	123 (3,6)	42 (11,9)	81 (2,7)	<0,001
Impaired functional status	628 (18,6)	265 (74,9)	363 (12)	<0,001
Institutionalized	492 (14,6)	148 (41,8)	344 (11,4)	<0,001

Media(m), Standard deviation (SD).

The mean heart rate of patients with dementia had a mean heart rate of 88.3 beats per minute, compared to 91.7 beats per minute in patients without dementia (p < 0.001). The mean arterial pressure was 86.4 mmHg in the dementia group and 89.2 mmHg in the non-dementia group (p < 0.001). The mean body temperature was 36.7°C in patients with dementia and 36.8°C in those without dementia (p < 0.001). The initial Glasgow score was 13.9 in the dementia group and 14.7 in the non-dementia group (p < 0.001), while the oxygen saturation was 88.3% and 89.1%, respectively (p < 0.001). FiO2 at admission was 28.1 in patients with dementia and 25.8 in those without dementia (p < 0.001).

Hypertension was present in 65.8% of the patients with dementia and 48.2% of those without dementia (p < 0.001). Smoking was reported in 30.5% and 20.8% of the patients, respectively (p < 0.001). Chronic heart failure was observed in 26.6% of the patients with dementia and in 14% of the patients without dementia (p < 0.001). The frequency of acute myocardial infarction was 9.3% in the dementia group and 5.2% in the non-dementia group (p = 0.002). Cerebrovascular disease was present in 20.9% and 7% of the patients, respectively (p < 0.001). Type 2 diabetes mellitus was reported in 16.1% of the patients with dementia and in 12% of the patients without dementia (p = 0.025). Chronic kidney disease was recorded in 11.9% and 4.3% of the patients, respectively (p < 0.001). Institutionalization was observed in 41.8% of patients with dementia and 11.4% of patients without dementia (p < 0.001). Altered functional status was present in 74.9% of the dementia group and in 12% of the non-dementia group (p < 0.001).

### Laboratory tests and imaging

The white blood cell count was 11.4 × 10³/L in patients with dementia and 12.6 × 10³/L in those without dementia (p < 0.001). Hemoglobin was 12.5 g/dL vs. 13.4 g/dL, respectively (p < 0.001). The hematocrit was 38.2% vs. 40.1% (p < 0.001). Platelet count was 242 × 10³/L vs. 260 × 10³/L (p < 0.001). Sodium level was 139.6 mmol/L and 137.3 mmol/L, respectively (p < 0.001). BUN was 30.6 mg/dL in the dementia group and 23.9 mg/dL in the non-dementia group, respectively (P < 0.001) (Table 2).

**Table 2:** Paraclinical examinations.

Paraclinical	Total Population n = 3374	Dementia n = 354	No dementia n = 3020	p Value
**Laboratory values, m (SD)**				
Leukocytes x10^3/ L	12,5 (6,4)	11,4 (6,0)	12,6 (6,4)	<0,001
Hemoglobin g/dl	13,3 (2,4)	12,5 (2,3)	13,4 (2,4)	<0,001
Hematocrit %	39,9 (6,9)	38,2 (6,8)	40,1 (6,9)	<0,001
Platelets x10^3/ L	258 (104)	242 (93)	260 (106)	<0,001
Sodium mEq/L	137,6 (6,2)	139,6 (8,5)	137,3 (5,9)	<0,001
Blood urea nitrogen mg/dl	24,6 (18,5)	30,6 (21,5)	23,9 (18)	<0,001
**Arterial blog gases, m (SD)**				
pH	7,41 (0,07)	7,40 (0,07)	7,42 (0,07)	<0,001
PaO₂	62,7 (19,7)	63,2 (20,75)	62,6 (19,55)	1,000
PaCO2	33,3 (8,61)	34 (8,18)	33,2 (8,66)	<0,001
HCO3	20,9 (4,2)	21,1 (4,3)	20,9 (4,2)	1,000
Lactate	2,1 (1,4)	2,3 (2,1)	2 (1,3)	<0,001
FiO2	29,7 (13,4)	30,6 (10,9)	29,6 (13,7)	<0,001
PaO2/FiO2 ratio	227,7 (71,5)	216,8 (69,4)	229,2 (71,7)	<0,001
**Chest X-ray, n (%)**				
Interstitial infiltrate	1686 (50)	232 (65,5)	1454 (48,1)	<0,001
Alveolar infiltrates	2301 (68,2)	292 (82,5)	2009 (66,5)	<0,001
Atelectasis	267 (7,9)	23 (6,5)	244 (8,1)	0,297
Consolidation	2241 (66,4)	282 (79,7)	1959 (64,9)	<0,001
Multilobar involvement	821 (24,3)	144 (40,7)	677 (22,4)	<0,001
Pleural effusion	514 (15,2)	53 (15)	461 (15,3)	0,004

Media(m), Standard deviation (SD), Partial pressure of oxygen (PaO2), Partial pressure of carbon dioxide (PaCO2) Bicarbonate (HCO3), Fraction of inspired oxygen (FiO2).

The pH was 7.4 in patients with dementia and 7.42 in those without dementia (p < 0.001). The partial pressures of carbon dioxide (PCO₂) were 34 and 33.2 mmHg, respectively (p < 0.001). Lactate reached 2.3 and 2 mmol/L (p < 0.001). PaO2/FiO2 ratio were 216.8 and 229.2 (p < 0.001), respectively. On chest radiographs, interstitial infiltrates were present in 65.5% and 48.1% (p < 0.001), consolidation in 79.7% and 64.9% (p < 0.001), and multilobar involvement in 40.7% and 22.4% of cases, respectively (p < 0,001).

### Clinical outcomes

The 30-day mortality rate was 9.1% (306/3374), with 26.3% in patients with dementia and 7.1% in those without (p < 0.001). The incidence rates of septic shock were 15% and 9.5%, respectively (p = 0.001) (Table 3).

**Table 3:** Clinical outcome.

	Total Population n = 3374	Dementia n = 354	No dementia n = 3020	p Value
**Clinical outcome n (%)**				
30-day mortality	306 (9,1)	93 (26,3)	213 (7,1)	<0,001
Septic shock	339 (10)	53 (15)	286 (9,5)	0,001
Vasopressor support	291 (8,6)	24 (6,8)	267 (8,8)	0,191
Intensive care unit (ICU)	430 (12,7)	31 (8,8)	399 (13,2)	0,017
Invasive mechanical ventilation	301 (8,9)	20 (5,6)	281 (9,3)	0,022
Non-invasive mechanical ventilation	152 (4,5)	16 (4,5)	136 (4,5)	0,989
Hospitalization Required	3032 (89,9)	342 (96,6)	2690 (89,1)	<0,001

Vasopressor support was required in 6.8% of the patients with dementia and 8.8% of those without dementia (p = 0.191). ICU admission rates were 8.8% and 13.2% (p = 0.017), respectively, and invasive mechanical ventilation was required in 5.6% and 9.3% of patients, respectively (p = 0.022). Non-invasive mechanical ventilation was used in 4.5% of patients in both groups (p = 0.989). Hospitalization was required in 96.6% and 89.1% of the cases, respectively (p < 0.001)

### Survival analysis

**Fig 2:**
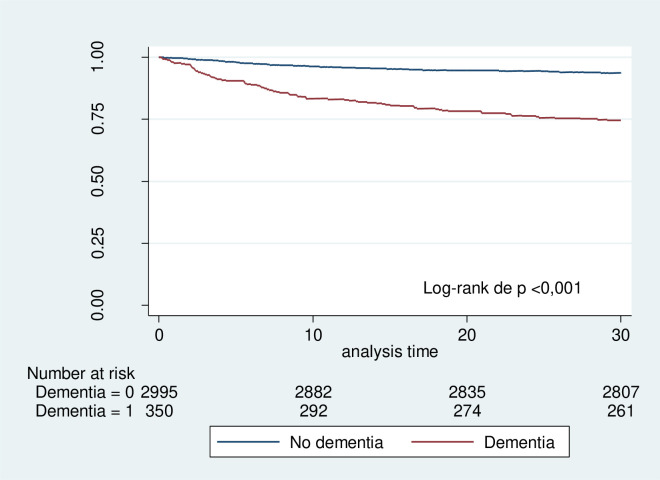
Kaplan-Meier survival estimates exclusion and inclusion criterio.

The 30-day survival rate in patients with community-acquired pneumonia without dementia was 92.9% compared to 73.7% in patients with dementia, showing a statistically significant difference by the log-rank test (p < 0.001) (Fig 2).

In Cox regression analysis, dementia showed a Hazard Ratio (HR) of 2.28 (95% CI: 1.756–2.962; p < 0.001) for 30-day mortality. The PSI score showed an HR of 1.02 (95% CI: 1.023–1.029; p < 0.001), and ICU admission was associated with an HR of 1.46 (95% CI: 1.091–1.967; p = 0.011) (Table 4).

**Table 4:** Risk factors for 30-day mortality in patients with pneumonia.

	HR	95% CI	p Value
Dementia	2,28	[1.756 - 2.962]	< 0,001
PSI score	1,02	[1.023 - 1.029]	< 0,001
Intensive care unit (ICU)	1,46	[1.091 – 1.967]	0.011

Pneumonia Severity Index (PSI).

p-Value were calculated using the Cox-proportional Hazard model.

## Discussion

In this study, dementia was identified as an independent mortality factor in patients with pneumonia (26.3% vs. 7.1%), with a Hazard Ratio of 2.28 (95% CI: 1.756–2.962; p < 0.001). Additionally, patients with dementia were older (82.5 years), had more comorbidities, worse initial clinical parameters, and greater pulmonary involvement, such as multilobar compromise. This group also showed a higher need for hospitalization and a higher prevalence of complications such as septic shock, reinforcing the association between dementia and poorer prognosis in this population.

The high mortality in patients with dementia is consistent with previous studies reporting an increased risk of death from pneumonia compared with individuals without dementia [6,14]. However, some studies have described similar mortality rates between the two groups [3], which could be attributed to differences in cohort characteristics, clinical management, access to intensive care, or outcome definitions. In our study, the higher mortality in patients with dementia could be explained by the high burden of comorbidities, clinical severity at admission, and advanced age observed in other investigations [15–17]. Furthermore, Mortensen et al. [18] indicated that factors such as elevated serum urea nitrogen levels, altered consciousness, and leukopenia present in our cohort are associated with higher mortality.

The relationship between pneumonia and dementia is complex and multiple factors. Systemic inflammation induced by respiratory infections can activate brain microglia, triggering a neuroinflammatory cascade that exacerbates neuronal damage [19,20]. Additionally, hypoxic stress associated with pneumonia can aggravate cognitive dysfunction by compromising critical areas, such as the hippocampus [21]. Long-term follow-up studies in patients who develop acute respiratory distress syndrome and severe COVID-19 have identified subsequent cognitive decline [22,23]. Although our study did not directly evaluate changes in cognitive function, the worse clinical outcomes and observed complications might suggest that these episodes accelerate the underlying neurodegenerative processes that worsen the patient’s condition, given the bidirectional relationship of these pathologies [19].

The initial clinical parameters observed, such as lower oxygen saturation and worse Glasgow scale scores, align with previous studies describing a more deteriorated functional status in patients with dementia hospitalized for pneumonia [17]. Furthermore, our study identified a higher prevalence of septic shock and multilobar pulmonary involvement, which are key factors in the clinical evolution of this population [15,16]. Moreover, the lower frequency of intensive care unit (ICU) admission in patients with dementia might reflect palliative care decisions, as suggested by some management approaches [24,25]. Additionally, Malecki et al. [26] emphasized the clinical heterogeneity within these patients in a subgroup analysis, which could influence therapeutic decisions and clinical outcomes.

Although our findings are consistent with those of previous studies, this study has several limitations. First, its retrospective design based on clinical records may introduce bias in data collection; however, the research team has experience in interpreting, extracting, and synthesizing this type of information. Second, the observational nature of the study could result in residual confounding due to unmeasured or unadjusted variables. Although we adjusted for potential confounders through multivariate analysis, the study’s design limits complete control over baseline differences. Third, while multiple clinical parameters were analyzed, important contextual factors—such as access to palliative care—were not assessed and could have influenced outcomes. Lastly, the limited detail and variability in patients’ clinical backgrounds emphasize the need for a deeper and more systematic understanding of baseline characteristics in future research, particularly in diverse and underserved populations.

## Conclusión

Patients with dementia who developed community-acquired pneumonia showed significantly higher mortality (HR 2.28, 95% CI: 1.756–2.962; p < 0.001). This population, characterized by advanced age, multiple comorbidities, and greater disease severity, highlights the need to implement specific and multidisciplinary clinical management strategies. Our findings underscore the importance of prioritizing individualized care for patients in our region, with the aim of improving their clinical outcomes and quality of life.
